# Current use of percutaneous ablation in renal tumors: an analysis of the registry of the German Society for Interventional Radiology and Minimally Invasive Therapy

**DOI:** 10.1007/s00330-024-11343-w

**Published:** 2025-02-28

**Authors:** Benedikt M. Schaarschmidt, Sebastian Zensen, Claudia Kesch, Thomas Dertnig, Marcel Opitz, Marcel Drews, Jonathan Nadjiri, Michael Forsting, Boris A. Hadaschik, Johannes Haubold

**Affiliations:** 1 Institute of Diagnostic and Interventional Radiology and Neuroradiology, University Hospital Essen, Essen, Germany; 2 Department of Urology, University Hospital Essen, Essen, Germany; 3 Department of Interventional Radiology, Klinikum rechts der Isar, Technische Universität München, Munich, Germany

**Keywords:** Kidney, Microwave ablation, Radiofrequency ablation, Cryoablation

## Abstract

**Objective:**

To evaluate the success and complications of thermal ablation (TA) based on the voluntary, prospective registry of the German Society for Interventional Radiology and Minimally Invasive Therapy (DeGIR) with 303 participating centers from Germany, Austria, and Switzerland.

**Materials and methods:**

Registry data from 2018 until 2023 of 1102 patients with small renal tumors (age: 72.5 ± 11.6 years; female: 33.6%, 370/1102) were analyzed. Hospitals with ≥ 20 TAs were considered high-volume centers. Technical success and complication rates between different parameters were compared using the chi-square or Fisher’s exact test, *p* < 0.05 was considered statistically significant.

**Results:**

Patients were most frequently treated with radiofrequency ablation (RFA, 43.6%, 481/1102), then microwave ablation (MWA, 41.9%, 462/1102) or cryoablation (13.3%, 147/1102). Technical success for heat-based TA (RFA&MWA) was 94.3% (893/947), for cryoablation 97.3% (143/147). RFA&MWA was significantly more successful in lesions ≤ 3 cm (96.1%, 567/590) compared to 3–4 cm lesions (89.8%, 97/108; *p* = 0.005). In patients treated with cryoablation, no significant differences between sizes could be found (≤ 3 cm: 97.9%, 94/96; 3–4 cm: 85.7%, 12/14; *p* = 0.078). Complication rate was significantly higher in RFA&MWA of lesions 3–4 cm compared to ≤ 3 cm (≤ 3 cm: 3.9%, 23/590; 3–4 cm: 11.1%, 12/108, *p* = 0.002), while no significant differences were seen regarding cryoablation (≤ 3 cm: 1.0%, 1/96; 3–4 cm: 0.0%, 0/14; *p* = 1.000).

**Conclusions:**

In this exploratory analysis of the DeGIR registry, percutaneous TA of small renal masses is technically feasible with low complication rates. Heat-based TAs seem to have lower success rates and higher complication rates in larger tumors. Cryoablation could potentially be a safe alternative for 3- to 4-cm-sized tumors.

**Key Points:**

***Question*** *How effective is renal thermal ablation (TA) in terms of treatment success and complication rates?*

***Findings*** *In contrast to cryoablation, heat-based thermal ablation has lower success and higher complication rates in tumors measuring 3–4 cm compared to tumors < 3 cm*.

***Clinical relevance*** *Thermal ablation is not influenced by the need for additional techniques such as cooling, protective organ displacement, or temporary vessel occlusion. For small renal tumors, TA is an effective and safe treatment option. Cryoablation could be beneficial in larger tumors.*

**Graphical Abstract:**

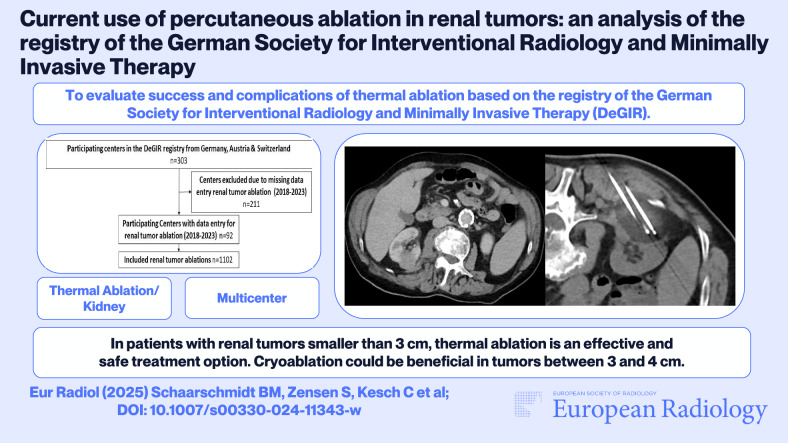

## Introduction

With an estimated number of 138,611 new cases in Europe, cancers of the kidney and the renal pelvis were in female patients the 11th, and in male patients the 6th, most common newly diagnosed cancer type in 2020 [1].

Surgical tumor removal is considered the treatment of choice in organ-confined renal cell carcinomas. with partial nephrectomy (PN) as the standard of care in tumors ≤ 7 cm (T1a and T1b) [2–4]. Alternatively, active surveillance or percutaneous thermal ablation (TA) can be offered for smaller renal masses (≤ 3–4 cm) [3, 4]. As some meta-analyses indicate higher overall survival rates as well as better local tumor control for PN than TA [5, 6], TA is mainly used in patients unfit for surgery or patients with impaired renal function or a solitary kidney [7, 8]. Furthermore, recent data on stereotactic body radiotherapy (SBRT) yielded promising results in the treatment of small renal masses [9, 10]. However, prospective evidence comparing these three treatments (PN, TA and SBRT) is not available, and most studies are based on retrospective analyses, which are prone to selection biases [5, 6, 11]. Furthermore, it remains unclear which ablative technique is best for the treatment of small renal tumors and thus the most promising alternative to PN. While heat-based TA relies on the destruction of tumorous tissue by heat generated either by a frequency alternating electric current (radiofrequency ablation, RFA) or microwaves (microwave ablation, MWA), cryoablation uses temperature reduction to induce cell death [12]. Initial studies indicated comparable results for heat-based TA and cryoablation alike [13, 14], but prospective studies are still lacking.

Analyzing clinical registries might provide important insights and help to design prospective studies exploring differences between the treatment approaches. The registry of the German Society for Interventional Radiology and Minimally Invasive Therapy (DeGIR) is of special interest. To constantly monitor the use of minimally invasive, image-guided therapies, the DeGIR encourages its members to share their experiences voluntarily in a prospective registry. Due to its broad user base with 303 participating centers from Germany, Austria and Switzerland and the availability of procedure-related data points, the data of this registry provides a valuable data source for previous studies [15, 16].

The aim of the present study was to evaluate indications, success and complications of TA for the treatment of renal cancer in central Europe exploring a large patient cohort (> 1000 patients) provided by this prospective registry.

## Materials and methods

For the present analysis, reports on percutaneous renal TA from 2018 until 2023 were obtained from the prospectively managed DeGIR registry (Samedi GmbH, Berlin, Germany). Here, participating centers can voluntarily submit data online for various image-guided interventions using an online form (supplementary data). For each year, data entry was possible until the end of February of the following year In the online form, the following parameters were considered mandatory: location of the tumor (upper pole/mid-section/lower pole), central tumor location (yes/no), emergency intervention (yes/no), outpatient procedure (yes/no), laboratory parameters (international normalized ratio (INR), partial thromboplastin time (PTT), platelet count, each indicated as normal, pathological or unknown), tumor etiology (metastasis, primary tumor, other, unknown), presence of difficult patient-related conditions, anesthesia, type of image guidance (CT/MRI/Angiography/Ultrasound), ablation technique, complications (before/after 24 h, type of complication, severity of complication), technical success (TA with safety margin > 5 mm, TA without safety margin, incomplete).

Complications were classified according to the Society of Interventional Radiology in minor (Grade A: no need for therapy, no consequences; B: Nominal therapy, no consequence; includes overnight admission for observation only) and major complications (C: Require therapy, minor hospitalization (< 48 h); D: Require major therapy, unplanned increase in level of care, prolonged hospitalization (> 48 h); E: Permanent adverse sequelae; F: death) by each participating center [17].

Other parameters were considered optional: therapeutic intent (palliative/curative), additional technique necessary, additional antibiotic treatment, unplanned termination of the intervention. Additionally, the ID of the participating center was registered for each patient. The database was prepared and checked for plausibility by a biostatistican (BA) prior to further analysis.

In a publication by Lu et al on RFA in liver tumors, the authors considered hospitals with more than 10 first-line treatments per year as high-volume centers. However, RFA of liver tumors is far more established than TA in renal tumors. Furthermore, the database grew over time, thus, not all centers participated over the whole time period. To ensure that high-volume centers that participated only in selected years were not falsely classified as low-volume centers, hospitals with ≥ 20 TAs during the analyzed time span were considered high-volume centers [18].

For statistical analysis, SPSS 23.0 (IBM) was used. Exploratory data analysis was performed for the three main types of TAs (RFA/MWA/cryoablation). Technical success and complication rates between different parameters were compared by the chi-square test, if not indicated otherwise. This analysis was considered to be exploratory, thus, alpha correction was not performed and *p* < 0.05 was considered as statistically significant.

The retrospective analysis of this registry was approved by the local ethics committee of the University Duisburg-Essen (Approval number: 23-11352-BO, date of approval: 25.07.2023).

## Results

### Patient characteristics

**Fig. 1:**
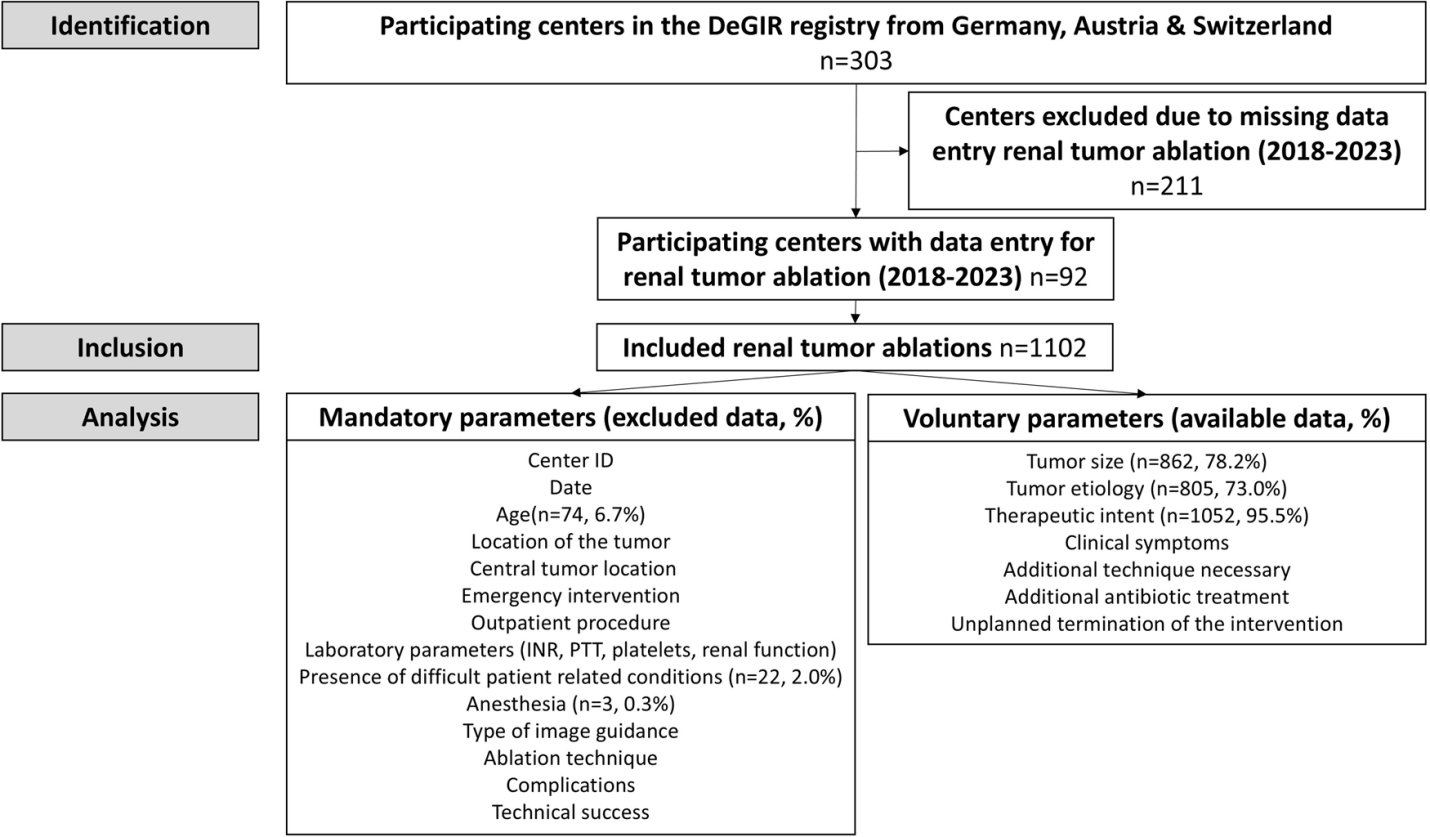
Study flow-chart depicting the process of patient identification, patient inclusion and data analysis of the DeGIR registry

From 2018 until 2023, percutaneous TAs of renal masses were performed in 1102 patients in 92 participating centers (Table 1, Fig. 1). A majority of patients (64.8%, 714/1102) were treated in high-volume centers (> 50 interventions: 41.5% (457/1102); 21–50 interventions: 23.3% (257/1102)). The other patients were treated in low-volume centers (11–20 interventions: 13.2%, 146/1102; 5–10 interventions: 13.3%, 147/1102; < 5: 8.6%, 95/1102, Table 2).

**Table 1:** Patient and tumor characteristics differentiated according to the various ablation methods

			Ablation technique		
		Total	RFA	MWA	Cryoablation
		*n* = 1102	43.6% (481/1102)	41.9% (462/1102)	13.3% (147/1102)
Sex	Female	33.6% (370/1102)	36.2% (174/481)	30.7% (142/462)	34.7% (51/147)
Male		66.4% (732/1102)	63.8% (307/481)	69.3% (320/462)	65.3% (96/147)
Age (years)		72.5 ± 11.6	72.6 ± 11.7	73.3 ± 11.0	71.0 ± 11.8
Tumor size (mm)		24.2 ± 11.2	24.9 ± 11.0	24.0 ± 11.6	22.4 ± 10.1
Tumor location	Right	44.9% (495/1102)	40.1% (193/481)	45.7% (211/462)	58.5% (86/147)
Left		41.0% (452/1102)	42.8% (206/481)	40.7% (188/462)	38.1% (56/147)
Both sides		0.6% (6/1102)	0.4% (2/481)	0.4% (2/462)	0% (0/147)
Not reported		13.5% (149/1102)	16.7% (80/481)	13.2% (61/462)	0.4% (4/147)
Upper pole		27.5% (303/1102)	25.6% (123/481)	30.1% (139/462)	27.2% (40/147)
Mid		40.7% (448/1102)	40.5% (195/481)	38.5% (178/462)	46.2% (68/147)
Lower pole		27.4% (302/1102)	28.7% (138/481)	26.4% (122/462)	25.9% (38/147)
Other		4.4% (49/1102)	5.2% (25/481)	5.0% (23/462)	0.7% (1/147)
Central tumor expansion		35.1% (387/1102)	27.4% (132/481)	43.9% (203/462)	32.0% (47/147)
Clinical symptoms		7.4% (82/1102)	3.7% (18/481)	8.2% (38/462)	16.3% (24/147)
	B symptoms	0.5% (5/1102)	0.6% (3/481)	0.4% (2/462)	0.0% (0/147)
	Hematuria	4.8% (53/1102)	0.6% (3/481)	6.1% (28/462)	10.9% (16/147)
	Pain	2.2% (24/1102)	1.5% (7/481)	1.7% (8/462)	5.4% (8/147)
Anemia		0.4% (4/1102)	0.6% (3/481)	0.0% (0/462)	0.7% (1/147)
Paraneoplastic syndrome		0.2% (2/1102)	0.2% (1/481)	0.2% (1/462)	0.0% (0/147)
Pathological laboratory parameters					
Creatinine		20.4% (225/1102)	21.6% (104/481)	17.5% (81/462)	26.5% (39/147)
PTT		2.9% (32/1102)	3.5% (17/481)	2.4% (11/462)	2.7% (4/147)
INR		4.0% (44/1102)	2.9% (14/481)	4.1% (19/462)	6.8% (10/147)
Platelet count		4.7% (52/1102)	3.1% (15/481)	5.4% (25/462)	8.2% (12/147)

**Table 2:** Frequency of percutaneous renal tumor ablations from 2018 to 2022

	2018	2019	2020	2021	2022	2023	Whole time span
Number of renal thermal ablations	148	180	175	162	148	289	1102
Change in frequency	N/A	+21.6%	−2.7%	−7.4%	−8.6%	+95.3%	N/A
(previous year)							
Participating centers	36	43	41	39	36	52	92
Type of ablation							
RFA	54.1% (80/148)	59.4% (107/180)	46.3% (81/175)	45.1% (73/162)	38.5% (57/148)	28.7% (83/289)	43.6% (481/1102)
MWA	40.5% (60/148)	37.8% (68/180)	48.6% (85/175)	50.0% (81/162)	50.0% (74/148)	32.5% (94/289)	41.9% (462/1102)
Cryoablation	3.4% (5/148)	1.7% (3/180)	5.1% (9/175)	4.3% (7/162)	9.5% (14/148)	37.7% (109/289)	13.3% (147/1102)
Other/not defined	2.0% (3/148)	1.1% (2/180)	0.0% (0/175)	0.6% (1/162)	0.7% (1/148)	0.3% (1/289)	0.8% (8/1102)
Multiple ablation types used	0.0% (0/148)	0.0% (0/180)	0.0% (0/175)	0.0% (0/162)	1.4% (2/148)	0.7% (2/289)	0.4% (4/1102)

### Lesion characteristics

Tumor etiology was reported in 73.0% (805/1102). The main treated tumor type was a primary renal tumor in 57.0% (628/1102), followed by metastases in 9.6% (107/1102) or other non-further specified tumors in 6.4% (70/1102).

Mean tumor size was 24.2 ± 11.2 mm (missing data: 21.8%, 240/1102) and lesions were nearly equally distributed between both sides (right: 44.9%, 495/1102; left: 41.0%, 452/1102). A central tumor expansion could be observed in 35.1% (387/1102). Clinical symptoms most likely associated with the treated kidney lesion were observed in 7.4% (82/1102) of all patients.

### Methods and technical approaches regarding percutaneous renal tumor ablation

**Fig. 2:**
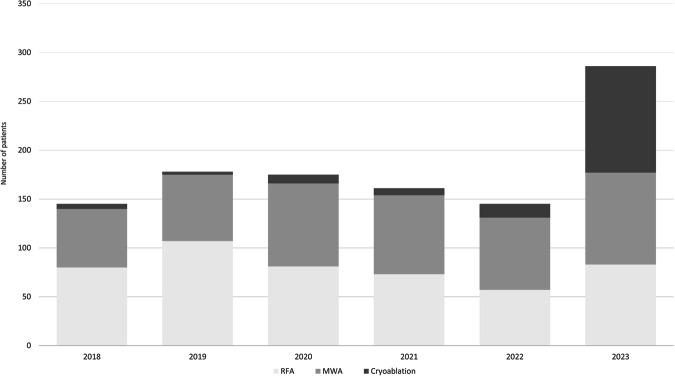
Use of different thermal ablation methods reported in the DeGIR registry from 2018 to 2023

**Fig. 3:**
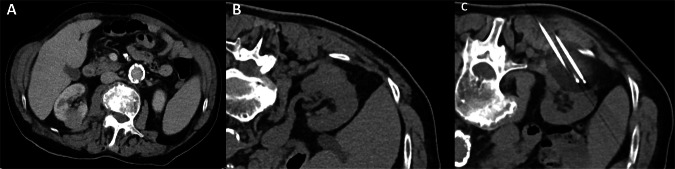
82-year-old patient with an incidentally detected renal mass in the right kidney (**A**, **B**). As the patient received anticoagulation due to endovascular aortic repair, cryoablation with a total of three cryoablation needles was successfully performed with a satisfactory safety margin (**C**)

The type of ablation was recorded for 1094 patients (Table 2). While an increase of documented renal TAs from 148 in 2018 to 180 in 2019 could be observed, we noticed a small but steady decline in the absolute number of renal TAs from 2020 to 2022, followed by an abrupt rise in 2023. Most patients were treated with RFA (43.6%, 481/1102), followed by MWA (41.9%, 462/1102) and Cryoablation (13.3%, 147/1102). A combined approach using RFA and MWA was performed in 0.4% (4/1102; missing data: 0.7%, 8/1102). While the number of performed MWAs showed a moderate increase over time (56.7%) and the number of RFAs nearly stagnated with a small increase of 3.8%, we observed a staggering increase of cryoablations from five cases in 2018 to 109 cases in 2023 (Figs. 2, 3).

Most procedures were performed with a curative intent (84.5%, 931/1102) while 11.0% were performed in a palliative setting (121/1102; missing data: 4.5%, 50/1102). Multiple lesions were treated in 8.2% (90/1102) of all reported interventions.

Renal TAs were mainly performed under CT guidance (97.3%, 1072/1102). Other imaging modalities such as fluoroscopic guidance (0.5%, 6/1102), ultrasound (0.5%, 6/1102) or MRI (0.2%, 2/1102) were rarely used. A combined approach using CT and ultrasound was performed in 1.1% (11/1102), CT and fluoroscopy were used in 0.4% (4/1102).

Renal TAs were mainly performed under general anesthesia (75.1%, 828/1102) or analgosedation (14.2%, 156/1102). Only selected interventions were performed under local anesthesia alone (7.7%, 85/1102) or sedation and local anesthesia (2.5%, 27/1102; other: 0.3%, 3/1102; missing data: 0.3%, 3/1102).

Difficult patient-specific conditions were observed in 29.4% (324/1102). These conditions included: difficult access route in 18.0% (198/1102), difficult local tumor situation in 14.7% (162/1102), obesity in 4.9% (54/1102), immobility in 3.1% (34/1102), risk of adverse reaction to contrast media in 1.2% (13/1102), cardiovascular instability in 0.5% (6/1102), and non-compliance in 0.5% (5/1102).

Additional techniques were performed in 23.1% (255/1102). Here additional cooling was performed in 15.8% (174/1102), protective organ displacement in 6.2% (68/1102) and temporary vessel occlusion in 0.4% (4/1102). Additional ablation of the needle track was performed in 58.6% (646/1102).

### Technical success rates of percutaneous renal tumor ablation

Renal TA could be completed in 99.6% (1098/1102) of cases. The intervention was terminated prior to complete TA either due to technical problems or intervention-related problems in two cases, respectively. Technically successful TA with a satisfactory safety margin could be achieved in 94.6% (1043/1102). In the remaining cases, TA was either performed without a satisfactory safety margin (3.4%, 37/1102) or was considered incomplete (2.1%, 23/1102).

Technical success for heat-based TA methods (RFA: 96.5% (464/481) and MWA: 92.0% (425/462), both methods combined: 100%: (4/4)) was 94.3% (893/947) and for cryoablation 97.3% (143/147). No significant difference was observed between the two groups according to a two-sided Fisher’s exact test (*p* = 0.166).

Size of renal lesions is considered an important predictor for technical success of TA. Treatment was less successful in lesions > 4 cm (85.7%, 7/49) than in lesions ≤ 4 cm (95.1%, 773/813; *p* = 0.01). Additionally, complete TA with safety margin was significantly less frequent for lesions between 3 and 4 cm (89.3%, 109/122) compared to lesions ≤ 3 cm (96.1%, 664/691; *p* = 0.001). Subgroup analysis revealed that this finding was most likely related to the high numbers of heat-based TAs: In patients treated with RFA&MWA, treatment was more successful in lesions ≤ 3 cm (96.1%, 567/590) compared to lesions between 3 and 4 cm (89.8%, 97/108; *p* = 0.005). In patients treated with cryoablation, however, no significant differences were found between lesions ≤ 3 cm (97.9%. 94/96) and lesions between 3 and 4 cm (85.7%, 12/14; *p* = 0.078 according to Fisher’s exact test).

Short-term treatment success was not significantly influenced by the need for additional techniques to perform a safe TA (additional technique necessary: 92.9%, 237/255; not necessary: 95.0%, 805/847; *p* = 0.195), center size (high-volume center: 94.8%, 677/714; low-volume center: 94.1%, 365/388; *p* = 0.602), impaired renal function (normal renal function: 95.4%, 754/790; pathologic renal function: 92.9%, 209/225; *p* = 0.125) or the presence of difficult patient-related conditions (present: 93.8%, 304/324; not present: 94.9%, 738/778; *p* = 0.492).

### Complication rates of percutaneous renal tumor ablation

Complications were reported in 4.8% (53/1102). Here, 2.5% (29/1102) were considered minor complications (SIR grade A and B) and 2.3% (25/1102) as major complications (SIR grade C-F) (Table 3). One patient suffered first from a minor venous bleeding and deceased later due to an ablation-related pulmonary complication (0.1%, 1/1102, Table 3). Most complications occurred during the first 24 h after the interventions (4.3%, 47/1102), six complications were observed 24 h after the intervention (0.5%, 6/1102).

**Table 3:** Complications of renal tumor ablations sorted by complications type

	Complication grade						Total
	Minor		Major				
	A	B	C	D	E	F	
Venous bleeding	3	8	0	1	0		1.1% (12/1102)
Arterial bleeding	0	3	7	0	1		1.0% (11/1102)
Parenchymal bleeding	5	2	1	2	0		0.9% (10/1102)
Pulmonary complications	1	3	2	0	0	1	0.6% (7/1102)
Infection	0	0	0	4	0		0.4% (4/1102)
Intestinal perforation	0	1	1	1	0		0.3% (3/1102)
Neurocerebral complications	0	0	1	0	0		0.1% (1/1102)
Other	3	0	2	1	0		0.5% (6/1102)
Total (according to complication grade)	1.0% (12/1102)	1.5% (17/1102)	1.9% (21/1102)	1.0% (11/1102)	0.4% (4/1102)	0.1% (1/1102)	
Total (according to complication grade)	2.5% (29/1102)		3.4% (27/1102)				

Severity of complications was rated according to the Society of Interventional Radiology [17]

Heat-based TAs had a complication rate of 5.4% (51/947), which was significantly higher than the complication rate of 1.4% (2/145) observed in patients treated with cryoablation (*p* = 0.034). However, no significant differences concerning major complications could be observed between the two treatment concepts (heat-based TA: 2.5%, 24/947; cryoablation: 0.7%, 1/147; *p* = 0.162).

While no significant difference was observed concerning complications according to Fisher’s exact test rates between lesions ≤ 4 cm (4.4%, 35/813) and lesions > 4 cm (8.2%, 4/45; *p* = 0.278), significant differences could be found between lesions ≤ 3 cm (3.5%, 24/691) and lesions measuring 3–4 cm (9.8%, 12/110; *p* = 0.002). In tumors treated with RFA&MWA, significantly more complications occurred in lesions with a diameter between 3 and 4 cm (11.1%, 12/108) than in lesions measuring ≤ 3 cm (3.9% 23/590; *p* = 0.002). In tumors treated with cryoablation, no significant differences were observed between the two groups (≤ 3 cm: 1.0%, 1/96; 3–4 cm: 0.0%, 0/14; *p* = 1.000 according to Fisher’s exact test).

Significantly more complications could also be observed in patients with impaired renal function (normal renal function: 4.1%, 32/790; pathologic renal function: 8.9%, 20/225; *p* = 0.004), in the presence of difficult patient-related conditions (present: 8.0%, 26/298; not present: 3.5%, 27/778; *p* = 0.0.003) and in patients treated in centers with lower volume centers (low-volume center: 7.7%, 30/388; high-volume center: 3.2%, 23/714; *p* = 0.0008). While no significant differences could be observed between high and low-volume centers concerning major complications (low-volume center: 2.8%, 11/388; high-volume center: 2.0%, 14/714; *p* = 0.352), the presence of difficult patient-related conditions was associated with a higher rate of major complications (present: 4.6%, 15/324; not present: 1.3%, 10/778; *p* = 0.0007).

Complication rate was not influenced by the need to perform additional techniques for a safe TA (additional technique necessary: 3.5%, 9/255; not necessary: 5.2%, 44/847; *p* = 0.320).

## Discussion

Image-guided ablation of renal tumors is a promising treatment option in localized disease. However, the lack of prospective studies, as well as the availability of various technical concepts, hinder a wide acceptance in the recent guidelines. This analysis of the DeGIR registry revealed four key findings. First, TA is a technically feasible treatment option for renal tumors with a low complication rate. Second, heat-based TAs have significantly lower success rates and higher complication rates in tumors between 3 and 4 cm compared to tumors smaller or equal to 3 cm. For cryoablation, however, no significant differences were observed here. Third, there is an increased adoption of cryoablation in 2023 in German speaking countries. Fourth, success and complication rates of TA are not influenced by the need for additional techniques to ensure a safe TA.

In the current guidelines, TA still plays only a minor role compared to PN due to the lack of high-level evidence. Especially European guidelines only recommend its use in patients with relevant comorbidities, patients with impaired renal function or a single kidney and patients with hereditary or bilateral tumors [4, 19]. The guidelines by the American Urological Association, however, consider percutaneous TA as an alternative to PN in small tumors [3]. The present analysis shows a high technical success rate of TA 94.6% in a large real-life cohort. These data are comparable to previously reported findings by Aarts et al (primary efficacy rate of 92% for RFA and 91% for MWA) [20] as well as Zhou et al and Abdelsalam et al (technical success rate of 100%, respectively) [21, 22], albeit our cohort included palliative procedures, where technical success was not the primary intent of the TA.

In the literature, complication rates between 11.5–20% [21, 23, 24] of which 3.0–5.5% were considered as major, were reported for TA [20, 22, 23]. Our study observed slightly lower complication rates, thus indicating an overall learning curve of interventional radiologists performing renal TA.

Renal tumor size is an important criterion to decide whether TA is an appropriate treatment form. As initial studies showed higher complication and local recurrence rates for tumors larger than 3 cm, most current urological and oncological guidelines recommend the use of TA only in tumors smaller than 3 cm [3, 19, 23]. A recent analysis of the Surveillance, Epidemiology, and End Results database from 2004 to 2018 detected a twofold higher cancer-specific mortality for heat-based TA compared to cryoablation in T1a tumors measuring 3–4 cm [25]. In our study, we observed a significant increase in complication rates and a decrease in technical success rates in tumors measuring 3–4 cm for heat-based TAs. In a subgroup analysis of patients treated with cryoablation, no significant differences could be found concerning technical success and complication rates. However, due to the small patient number of the 3–4 cm subgroup (*n* = 14) the statistical power of this observation is limited. The steady rising share of cryoablations should allow further analysis with a bigger cohort in the future. Hence, our findings support the recommendations of the Society of Interventional Radiology and the European Association of Urology which favor the use of cryoablation in renal tumors of 3–4 cm size [26].

The advantages of cryoablation are also reflected by its increased use. In the past, RFA was used more frequently than cryoablation in Germany while cryoablation was more often used in the US [27]. The present data indicate that after the COVID-19 pandemic, there was a staggering increase in cryoablations in central Europe while the relative proportion of heat-based TAs diminished.

Initially, posterior or posterolateral tumors were considered ideal candidates for percutaneous TA to avoid damage to adjacent structures such as ureter, bowel or vessels [28]. However, the development of new adjuvant techniques such as organ displacement via air or hydrodissection [21, 23, 29], additional cooling [21, 23] or even tumor vessel occlusion [30] have increased the number of renal tumors considered treatable by percutaneous TA considerably. In our analysis, adjuvant procedures for renal TA were reported in 23.1%, which is comparable to rates of 16.5–39% in the literature [21, 23]. Interestingly, the need for adjuvant procedures did neither have a significant impact on technically successful tumor treatment nor complication rate.

Center volume is considered an important predictor for treatment success and complications. Especially for RFA of hepatocellular carcinomas of the liver, low center volumes are attributed with a higher in-hospital mortality [31] and lower overall survival rates [18]. These observations cannot be translated to renal tumors. Although higher complication rates were observed in small volume centers, these did neither translate to an increased rate of major complications nor a decreased technical success rate. Therefore, TA in renal tumors might be possible not only in a few selected high-volume centers, but could be made available to many patients in local tumor treatment facilities. However, further research concerning the medium and long-term oncological outcome is necessary to evaluate the impact of center volume on TA in renal tumors.

Our study has limitations related to the design of the DeGIR registry which works on a voluntary basis. Hence, a selection bias is possible as experienced centers with less complications are expected to be more interested in sharing their results. As many parameters are optional and central monitoring is not performed, a reporting bias cannot be excluded. Furthermore, important parameters such as tumor size, treatment success and complication grade were entered into the database by the participating centers without reevaluation by a centralized imaging core laboratory. Furthermore, long-term outcome data are missing. Moreover, treatment benefit was evaluated only by using technical success as a surrogate parameter. Data on recurrence-free or cancer specific survival were not available but would be beneficial in the context of treatment success.

To minimize the workload for participating centers, the data points are carefully selected to ensure a swift input of all necessary data. Therefore, selected factors such as the differentiation between an anterior or posterior location of a renal tumor as well as imaging follow-up and survival data are not included in the present dataset [8]. Still, the analysis of the DeGIR registry provides important real-life, multicenter data on TA in renal tumors in a large, prospective cohort.

## Conclusions

Percutaneous TA of renal tumors is a safe intervention with a low complication rate especially in tumors < 3 cm. While a drop in reported interventions could be observed during the COVID-19 pandemic, an increase in interventions could be observed in 2023 with a steady rising share of cryoablations which is potentially advantageous in tumors between 3 and 4 cm.

## Supplementary information

ELECTRONIC SUPPLEMENTARY MATERIAL

## Abbreviations

DeGIR: German Society for Interventional Radiology and Minimally Invasive Therapy
MWA: Microwave ablation
PN: Partial nephrectomy
RFA: Radiofrequency ablation
SBRT: Stereotactic body radiotherapy
TA: Thermal ablation
