# Supplementary material for: Current use of percutaneous ablation in renal tumors: an analysis of the registry of the German Society for Interventional Radiology and Minimally Invasive Therapy
Source: Eur Radiol. 2025 Feb 28;35(3):1723–31. doi: 10.1007/s00330-024-11343-w (PMC11868359; doi:10.1007/s00330-024-11343-w)
Supplement: Supplementary file 1 — ELECTRONIC SUPPLEMENTARY MATERIAL [file 330_2024_11343_MOESM1_ESM.pdf]

Current use of percutaneous ablation in renal tumors: an analysis of the registry of the German Society for Interventional Radiology and Minimally Invasive Therapy

ELECTRONIC SUPPLEMENTARY MATERIAL

Online form for data submission for image-guided interventions for the DeGIR registry (Samedi GmbH, Berlin, Germany).

*Mandatory questions are marked with *. Facultative questions are given in italic. Answering options are given in brackets.*

Patient data

Sex * (male; female; diverse)
Date of birth *

1 - Intervention

Date*
Region* (Kidney)
Target detail* (Upper pole, middle, lower pole)
Central?* (yes; no)

Interventionalist

Interventionalist 1
Interventionalist 2
Interventionalist 3

Outpatient intervention?* (yes; no)
Begin of intervention outside routine daily shift * (yes; no)

2 - Preinterventional diagnostic

Therapeutic intention: (curative; pallative)

Lab: *

Quick/INR* (n.a.; normal; pathological)
PTT* (n.a.; normal; pathological)
Thrombocyte count* (n.a.; normal; pathological)
Creatinine/GFR* (n.a.; normal; pathological)

Preinterventional imaging *

X-ray (ex. mammography) (yes)
Mammography (yes)
Tomosynthesis (yes)
CT/CTA (yes)
MRI/MRA (yes)
DSA (yes)
Ultrasound (yes)
Scintigraphy/SPECT/SPECT-CT (yes)
PET/PET-CT/PET-MRI (yes)
not done (yes)

Clinical indication *

Presurgical procedure? (yes; no)
Metastasis? (yes)
Primary tumour? (yes)
Osteoidosteoma/Osteoblastoma? (yes)
Uterusmyoma? (yes)
Other (yes)
Unknown (yes)

Lesion size (mm)

Clinical symptoms (yes; no)

if yes: (Pain, Pseudocyst, Hematuria, Anemia, paraneoplastic syndrome, B-symptoms)

Interdisciplinary conference *

Anaesthesiology (yes)
General surgery/ Vascular surgery (yes)
Internal medicine (yes)
Neurosurgery (yes)
Neurology (yes)
Radiology (yes)
Pathology (yes)
Radiation therapy (yes)
Nuclear medicine (yes)
Urology (yes)
Others (yes)
Conference decision (yes)
Not done (yes)

3 - Descrption of intervention

Anaesthesia *

No (yes)
Local (yes)
Sedation (yes)
Analgosedation (yes)
Intubation (yes)
High-flow ventilation (yes)
Anaestesiological stand-by (yes)
Other (yes)

Medication

Antibiotic treament (yes; no)

Method of ablation *

RFA (yes)
Laser (LTT) (yes)
MWA (yes)
Cryoablation (yes)
Focused ultrasound (yes)
Irreversible Elektroporation (IRE) (yes)
Electrochemotherapy (ECT) (yes)
intraoperative (yes)
other (yes)

Additional method

Additional ablation of the needle track (yes)
Protective organ displacement (e.g. using gas or liquid) (yes)
cooling (yes)
Temporary vascular occlusion (e.g. balloon occlusion) (yes)

Complexitiy of intervention

Number of lesions *
Needle positions *

4 - Quality of results

Image-guided technique *

CT (yes)

CTDI vol* DLP*

DSA (yes)

Cone beam CT (yes; no)
Duration
DAP

MRI (yes)
Ultrasound (yes)
Mammography (yes)

Difficult conditions *

No (yes)
Obesity (yes)
Non-compliance (yes)
ventilated patient (yes)
Increased risk for use of contrast agent (yes)
Cardiovascular instability (yes)
Immobilized patient (yes)
difficult access (yes)
difficult local tumor situation (yes)
need of special technique (yes)
other (yes)

Technical success

Technical success* (yes, no, partial success)

Clinical success early post-interventional

Clinical success early post-interventional* (yes; no; not evaluable)

Postinterventional imaging*

no imaging (yes)
X-ray (excl. mammography) (yes)
Mammography (yes)
CT/CTA (yes)
MRI/MRA (yes)
DSA (yes)
Ultrasound/Duplex (yes)
Szintigraphy/SPECT/SPECT-CT (yes)

PET/PET-CT/PET-MRI (yes)

Complications Module A-D

Complications Module A-D within 24h post intervention * (yes; no)

if yes: Complication (arterial bleed, venous bleeding, arterial occlusion, venous occlusion, aneurysm, cardiac complication, neuro-cerebral complication, pulmonary complication, infection/abscess, dislocation of external material [primary or secondary], parenchymal bleeding, tissue ischemia or infarction, anaphylaxia, organdysfunction , -failure, adverse medication reaction, organ injury, other)

Complication treated? (no; self; other discipline)

if yes: Success? (yes; no)

Complications Module A-D after 24h post intervention * (yes; no)

Termination of intervention (yes; no)

if yes: Reason: technical/anatomical reason, incompliance of patient, complication independant of the intervention, complication associated with the intervention, technical failure of the machine, probing of the lesion not successfull, other)

Multiple interventions? (yes)
